# Awareness and opinions regarding contraception by women of reproductive age in North-West Nigeria

**DOI:** 10.11604/pamj.2018.30.65.12975

**Published:** 2018-05-28

**Authors:** Adewole Adebola Adefalu, Oladapo Alabi Ladipo, Oluwaseun Oladapo Akinyemi, Oluwafemi Akinyele Popoola, Olajimi Oluwatosin Latunji, Omowunmi Folake Iyanda

**Affiliations:** 1 Association for Reproductive and Family Health, Abuja, Nigeria; 2 Department of Health Policy and Management, College of Medicine, University of Ibadan, Nigeria; 3 Department of Community Medicine, College of Medicine, University of Ibadan, Nigeria

**Keywords:** Maternal mortality, contraceptive uptake, North-west Nigeria

## Abstract

**Introduction:**

North-west Nigeria is a traditionally high fertility setting. Increasing attention is being channeled into scaling up the various interventions that can reduce high fertility, including enhancing contraceptive uptake among women of reproductive age. However, in order to improve demand for contraception, understanding the level of awareness, knowledge and perception of WRA to contraceptive use is essential. This study examines the level of knowledge and perception of WRA to contraceptive use.

**Methods:**

A descriptive cross-sectional study was carried out in December 2016 in Kebbi and Sokoto states, North-west Nigeria. Semi-structured questionnaires were administered to 500 women between the ages of 15-49 years in 4 randomly selected local government areas across the states. Data analysis included descriptive and bi-variate statistics.

**Results:**

Only 43.8% claimed to be currently using a modern form of contraception; 82.4% were aware of at least one form of contraception, while health personnel formed the major source of information. The most popular and least common modern contraceptive methods were the male condom (86.8%) and vasectomy (26.6%) respectively. A higher proportion of married respondents (88.8%) than those presently single (68.8%) had good knowledge of contraceptives. Perception of contraceptives was mixed, with majority believing that contraceptives should be made available to every woman, despite stating that it contradicted their religious beliefs.

**Conclusion:**

These results underline the need for policy makers to focus on improving the knowledge of younger age groups on contraceptives and include other information channels such as peer-to-peer discussions to increase awareness.

## Introduction

A breakdown of the global health statistics shows that every year, over 40,000 women in Nigeria die from childbirth and complications arising from pregnancy [1]. Thus, while Nigeria is responsible for only 2.5% of the world's population, it is responsible for 14% of maternal deaths globally every year [1]. These appalling statistics has led to increased interest in improving maternal health in Nigeria. However, in spite of this interest, not much has been achieved in terms of improving maternal health in the country over the past 15 years [2]. For example, while the 2008 National Demographic and Health Survey (NDHS) reports a maternal mortality rate of 545 per 100,000 live births, the 2013 NDHS reports 576 per 100,000 live births, an increase of 5.6% [3,4]. This contrasts with both the Millennium Development Goal (MDG 5) of 75% reduction in maternal mortality rates and the Sustainable Development Goal (SDG 3) respectively. In order to reverse this negative trend, stakeholders have identified that improving contraceptive uptake among women of reproductive age is critical to any progress Nigeria is to record [5,6]. This is because contraceptive use has been observed to delay onset of child bearing as well as reduce parity among users. In turn, research has shown that such women are less likely to die from childbirth. However, the latest NDHS reports that only 10% of women currently use a modern form of contraceptive. This figure is further skewed by the North-South divide with the North-west particularly recording worse contraceptive use statistics than other regions in the country [3]. Thus, improving contraceptive uptake among women of reproductive age (WRA) in North West Nigeria is a critical component of improved maternal health in the country [7,8]. Literature has shown that there are 2 main drivers of contraceptive uptake, namely demand and supply [9]. The supply side, which involves the provision of contraceptives at convenient points in order to reduce and if possible eliminate unmet need has been given much attention by both government and non-governmental organizations [10,11]. However, the demand side for contraceptive demonstrates the willingness to use contraceptives, which will in turn inform decisions about supply [12]. The demand side of contraceptive uptake has received a measure of attention from government, donor programs and other non-governmental organizations through activities such as social mobilization, mass media and other slogans and logos worn at public events [7]. The aim of these activities is to foster dialogue about family planning, increase social approval for family planning and improve knowledge and perceptions of family planning methods [13,14]. While literature has identified socio-cultural factors such as religion, culture and fear of side effects as potential influencers of contraceptive uptake in the region, there is still a paucity of studies that focus on specific perceptions of WRA to contraceptive uptake in the Northwest region of Nigeria. This study aims to provide this information by investigating the awareness, knowledge and perceptions of WRA in the region.

## Methods

**Study sites**: The research was conducted in two states in North-West Nigeria, namely Sokoto and Kebbi States. Sokoto State lies to the north-west of Nigeria and shares its borders with Niger Republic to the North, Katsina State to the East, Niger State to the South-East, Kwara State to the South and Benin Republic to the West. Sokoto State consists of twenty-three (23) Local Government Areas (LGA). Sokoto state is ranked 14^th^ in Nigeria based on the population of each state. Kebbi State was created out of a part of Sokoto State in 1991. Kebbi State is bordered by Sokoto State, Niger State, Zamfara State, Dosso Region in the Republic of Niger and the nation of Benin. It has a total area of 36,800 km^2^. The State has a total population of 3,137,989 people as projected from the 1991 census, within 21 LGAs. The people of Kebbi are predominantly Muslims who practice Islam.

**Study design and data collection tool**: A descriptive, cross-sectional study was carried out in December 2016 among women between the ages of 15 and 49 years in the selected LGAs of Kebbi and Sokoto states. A semi-structured questionnaire consisting of closed and open ended questions was developed and used to generate useful information that addresses both the dependent and independent variables of the subject matter of the survey. The questionnaire was divided into different sections for easy response filling and in a chronological order. The questionnaire was developed in a user-friendly manner such that it can be self-administered and interviewer administered by literate and illiterate respondents respectively. The instrument was translated into Hausa language and then back-translated to English buy an independent translator to ensure that accuracy of thought was maintained.

**Sample size calculation**: Using the total unmet need for family planning among women of reproductive age (15-49) (NDHS, 2013), 16% in Kebbi State, the sample size was estimated using the Leslie Kish formula for sample size calculation at 95% confidence interval and 20% attrition/non-response rate.

**Sample size calculation**: The sample size was estimated using the Leslie Kish formula (1965) shown below:

$$\text{N=}\frac{{\left(Z\text{α}\right)}^{2}P\text{q}}{{\text{d}}^{2}}$$

Where N is the minimum sample size Zα = standard normal deviate corresponding to level of significance; 1.96 at α (Type 1 error) = 5% P= Estimated unmet need for family planning in Northwest Nigeria (NDHS, 2013) at 16% = 0.16 q= 1-P = (1-0.16) = 0.84 d= desired level of precision = 5% = 0.05 (maximum sampling error allowed)

$$\begin{array}{l} \text{N}=\frac{{\left(1.96\right)}^{2}\times 0.16\times 0.84}{0.05^{2}}=206.5\\ \text{Approximated to 207} \end{array}$$

Anticipating 20% non- response rate (nr), the sample size = N × 1/1-nr 207 × 1/1-0.2 = 248.75. Thus, a minimum sample size of 497 respondents for the 2 states was calculated. This was approximated to 500 respondents.

**Sampling technique and sample selection**: All the total wards in each of the selected LGAs were involved in the survey. With support of the community persons, house listing and marking were conducted to determine the number of houses in each of the wards. Systematic sampling technique (in direct proportion to the number of houses in the respective settlements) was used to determine the households for recruitment of participants. This was done until the required sample size was obtained.

**Independent and dependent variables**: The independent variables included socio-demographic characteristics such as age group, sex, religion, family type, settlement type, parity etc. Dependent variables included awareness of contraceptives categorized into 2 (aware or unaware) and knowledge about contraceptives which was categorized into 2 (good knowledge or poor knowledge).

**Instrument validity**: Content validity was achieved through review of literature to develop a pool of questions for the survey while the draft questionnaire was subjected to face validation by experts in the field of community medicine. . The instrument was finally pretested among 50 WRA (10% of the sample size) in Anka LGA of Zamfara State. Cronbach Alpha scores of 0.81 indicated high reliability of the instrument.

### Data management

**Data collection procedure and implementation**: Recruitment and selection of interviewers was carried out using selection criteria such as willingness to work, ability to read and write in English and Hausa language, familiarity with the geographical and household setting of the data collection site, good communication skills among others. A one-day orientation and training was conducted to increase capacity of the research assistants for effective implementation. The training was to ensure that the research assistants were acquainted with the project goal, objectives and strategy, the survey methodology and data collection tools. The training also involved lectures, discussions, as well as demonstration and return demonstrations with the technical supervision of the research team to assure full understanding of the methodology.

**Data analysis**: Data were entered, sorted, cleaned and analysed using Statistical Package for Social Sciences (SPSS) version 21 software. Scores were allotted to required questions with correct answers scored as 1 and wrong answers scored as 0. Based on this, respondents with average scores of 50% and higher were classified as having good knowledge while respondents with less than 50% were classified as having poor knowledge. Descriptive statistics such as proportions and frequencies were used to describe the data while chi-square tests were carried to test for statistical associations between dependent and independent variables at 5% level of significance. Chi-square statistic and p-values were recorded for the chi-square tests.

**Ethical considerations**: Ethical approval was granted by the Ethics Committee of Association for Reproductive and Family Health. Permission to conduct the study was granted by local community leaders, local government officials and other stakeholders. Written informed consent was also obtained from the clients and stakeholders prior to questionnaire administration and respondents' anonymity were protected by ensuring that no individual identifiers existed in the instruments or in the electronic data set. Confidentiality of data was ensured by keeping the data on a password-protected laptop with only research staff having access to the password.

## Results

The majority of respondents (84.0%) were married while a similar proportion (84.2%) of respondents were Muslims. The educational attainment of the respondents was more evenly distributed with 14.2% of respondents having completed no form of formal education at all and 13.4% of the respondents completed tertiary education. Less than half (43.8%) of the respondents claimed to be currently using a modern form of contraception. Table 1 shows the comprehensive sociodemographic characteristics of the respondents.

**Table 1:** Sociodemographic characteristics of the respondents (N=500)

	N	%
**Marital Status**		
Presently Single	80	16.0
Married	420	84.0
**Age Group**		
15-19	15	3.0
20-24	53	10.6
25-29	127	25.4
30 and older	305	61.0
**Religion**		
Christianity	75	15.0
Islam	421	84.2
Others	4	0.8
**Ethnicity**		
Yoruba	42	8.4
Igbo	45	9.0
Hausa	382	76.4
Others	31	6.2
**Education**		
None	71	14.2
Basic	362	72.4
Tertiary	67	13.4
**Family Type**		
Monogamous	272	54.4
Polygamous	228	45.6
**Contraceptive Uptake**		
Currently using a modern method	219	43.8
Not currently using a modern method	281	56.2
**Settlement**		
Rural	147	29.4
Semi-urban	79	15.8
Urban	274	54.8
**Parity**		
0-4	255	51.0
More than 4	245	49.0
**Local Government Area**		
Argungu	100	20.0
Aliero	150	30.0
Sokoto North	119	23.8
Sokoto South	131	26.2

**Awareness of contraceptives**: Table 2 shows that 82.4% of the respondents were aware of contraceptives while health personnel were the main source of information on contraceptives for about two-thirds (66.4%) of the respondents who were aware of contraceptives. Among the total respondents, the most popular function of contraceptives remained child spacing (85.8%) while a little more than three-fifths of the respondents (63.4%) knew that contraceptives could be used to prevent unwanted pregnancy. The least known function (25.2%) of contraceptives among the respondents was limiting family size. The most popular modern contraceptive method was the male condom (86.8%) followed closely by the diaphragm (81.0%), while the diaphragm (28.2%) and vasectomy (26.6%) were the least known modern contraceptives. Results from the chi-square analysis revealed that the difference between the proportions of married people and single people aware of contraceptives was not statistically significant. (p=0.115). With regard to educational attainment, it was discovered that the higher the educational level completed, the higher the proportion of members that were aware of contraceptives. Table 3 also shows that this association was statistically significant (p<0.001). However, it was noted that while awareness was highest in the urban areas, awareness in the rural areas was actually higher than in semi-urban areas. The difference between these proportions was statistically significant (p < 0.001).

**Table 2:** Awareness of contraceptives (N=500)

	n	%
**Awareness on contraceptives**		
Aware	412	82.4
Unaware	88	17.6
**Main Source of Information (N=221)**		
Spouse	41	10.0
Health personnel	273	66.4
Mass media	20	4.9
Friends	77	18.7
Uses of Contraceptives (N= 250)*		
Preventing unwanted pregnancy	317	63.4
Child spacing	429	85.8
Limiting family size	126	25.2
Known contraceptive methods (N= 250)*		
Male condom	434	86.8
Female condom	405	81.0
Diaphragm	141	28.2
Injectable	295	59.0
IUCD	294	58.8
Oral contraceptives	310	62.0
Implant	365	73.0
Tubal ligation	178	35.6
Vasectomy	133	26.6
Emergency pills	201	40.2
Others	118	23.6

* - Multiple response variable

**Table 3:** Bivariate analysis of factors associated with awareness of contraceptives

	Awaren (%)	Unawaren (%)	X2	p-value
**Marital Status**				
Single/Presently Unmarried	61 (76.3)	19 (23.8)	2.48	0.115
Married	351 (83.6)	69 (16.4)		
**Age Group**				
15-19	9 (60.0)	6 (40.0)	6.86	0.077
20-24	41 (77.4)	12 (22.6)		
25-29	105 (82.7)	22 (17.3)		
30 and older	257 (84.3)	48 (15.7)		
**Religion**				
Christianity	67 (89.3)	8 (10.7)	9.08	0.049
Islam	343 (81.5)	78 (18.5)		
Others	2 (50.0)	2 (50.0)		
**Ethnicity**				
Yoruba	41 (97.6)	1 (2.4)	9.31	0.025
Igbo	39 (86.7)	6 (13.3)		
Hausa	309 (80.9)	73 (19.1)		
Others	23 (74.2)	8 (25.8)		
**State**				
Kebbi	221 (88.4)	29 (11.6)	12.41	<0.001
Sokoto	191 (76.4)	59 (23.6)		
**Education**				
None	48 (67.6)	23 (32.4)	25.06	<0.001
Basic	297 (82.0)	65 (18.0)		
Tertiary	67 (100.0)	0 (0.0)		
**Family Type**				
Monogamous	230 (84.6)	42 (15.4)	1.92	0.166
Polygamous	182 (79.8)	46 (20.2)		
**Settlement**				
Rural	122 (83.0)	25 (17.0)	21.78	<0.001
Semi-urban	51 (64.6)	28 (35.4)		
Urban	239 (87.2)	35 (12.8)		
**Parity**				
0-4	217 (85.1)	38 (14.9)	2.61	0.106
More than 4	195 (79.6)	50 (20.4)		

**Knowledge on contraceptives**: Knowledge on contraceptives was high among the respondents with 428 (85.6%) having good knowledge on contraceptives. A further break down of the data, as shown in Table 4, revealed that while 88.8% of married respondents had good knowledge about contraceptives, only 69.2% of presently unmarried respondents had good knowledge. This association was found to be highly statistically significant (p < 0.001). Other independent variables that were found to be associated and statistically significant with knowledge about contraceptives were age group (p < 0.001), education (p=0.003) and ethnicity (p = 0.025).

**Table 4:** Factors affecting knowledge about contraceptives

	Poor Knowledge n (%)	Good Knowledgen (%)	X2	p-value
**Marital Status**				
Single/Presently Unmarried	25 (31.3)	55 (68.8)	21.94	<0.001
Married	47 (11.2)	373 (88.8)		
**Age Group**				
15-19	8 (53.3)	7 (46.7)	23.37	<0.001
20-24	12 (22.6)	41 (77.4)		
25-29	16 (12.6)	111 (87.4)		
30 and older	36 (11.8)	269 (88.2)		
**Religion**				
Christianity	8 (10.7)	67 (89.3)	3.35	0.501
Islam	63 (15.0)	358 (85.0)		
Others	1 (25.0)	3 (75.0)		
**Ethnicity**				
Yoruba	4 (9.5)	38 (90.5)	9.31	0.025
Igbo	5 (11.1)	40 (88.9)		
Hausa	10 (32.3)	21 (67.7)		
Others				
Sokoto				
**Education**				
None	9 (12.7)	62 (87.3)	11.41	0.003
Basic	62 (17.1)	300 (82.9)		
Tertiary	1 (1.5)	66 (98.5)		
**Family Type**				
Monogamous	42 (15.4)	230 (84.6)	0.53	0.469
Polygamous	30 (13.2)	198 (86.8)		
**Settlement**				
Rural	28 (19.0)	119 (81.0)	4.96	0.084
Semi-urban	13 (16.5)	66 (83.5)		
Urban	31 (11.3)	243 (88.7)		
**Parity**				
0-4	42 (16.5)	213 (83.5)	1.81	0.178
More than 4	30 (12.2)	215 (87.8)		

**Perception about contraceptives**: More than half of the respondents (57.8%) agreed with the notion that family planning (FP) conflicts with their moral/cultural/religious beliefs while only 36.6% of them agreed that FP conflicts with their moral/cultural/religious beliefs. In addition, 62.5% of the respondents disagreed that FP would make users more promiscuous while almost one-quarter (82.2%) of them agreed with the notion that men should be involved in family planning issues. More than two out of every three respondents (69.2%) disagreed that contraceptives are harmful because of their side effects. Table 5 shows the perception of the respondents to contraceptives.

**Table 5:** Perception of respondents towards contraceptives

	Agreen (%)	Undecidedn (%)	Disagree/n (%)
Family planning/contraceptive conflict with my moral/cultural/religious beliefs	289 (57.8)	28 (5.6)	183 (36.6)
All women of reproductive age should have access to contraceptives /family planning service	383 (76.6)	27 (5.4)	90 (18.0)
Family planning is beneficial to the society	424 (84.8)	54 (10.8)	22 (4.4)
Family planning is beneficial to women only	176 (35.2)	71 (14.2)	253 (50.6)
Family planning is beneficial to men	319 (63.8)	78 (15.6)	103 (20.6)
Family planning is beneficial to children	412 (82.4)	19 (3.8)	69 (13.8)
Family planning is not good for both man and woman	85 (17.0)	37 (7.4)	378 (75.6)
Family planning/contraceptives make users promiscuous	129 (25.8)	59 (11.8)	312 (62.4)
Contraceptives harmful because of side effects	108 (21.6)	46 (9.2)	346 (69.2)
Contraceptives are actually effective in preventing pregnancies	442 (88.4)	25 (5.0)	33 (6.6)
Men should be involved in family planning issues	411 (82.2)	23 (4.6)	66 (13.2)
Family planning services/contraceptives service is too expensive	41 (8.2)	33 (6.6)	426 (85.2)

## Discussion

The level of contraceptive uptake was higher than has been reported in other reports and in the NDHS [15]. The fact that most of the respondents were from urban areas could have played an important part in increasing the level of contraceptive uptake as few of the respondents came from the rural areas. Other studies in urban areas also report higher contraceptive prevalence rates than studies carried out in both urban and rural settings [7,16] Awareness and knowledge of FP and contraceptives were found in this study to be very high indicating that the majority of the respondents had heard of contraceptives. Studies have identified awareness of contraceptives as the important first point in the continuum that leads to contraceptive uptake [6,15,17]. A notable finding of this study is that an appreciable portion of respondents cited friends as their main source of information on contraceptives. Thus, the peer-to-peer transmission route appears to be an important one in the dissemination of information about FP and contraceptives in the state. Despite the high level of awareness reported in this study, differentials still point to skewed distribution, exposing factors that affect the level of knowledge of different segments of a population about FP and contraceptives. Similar differentials were observed in other studies carried out in the same region [5,18]. Urban residents and those completing tertiary education were found to be more likely to be aware of contraceptives than their counterparts living in semi-urban areas and have no formal education. This uneven level of awareness and knowledge among different groups in the population may have the potential to hamper efforts to increase FP uptake if not properly addressed [9]. Also, there was an inverse relationship between knowledge and age of the respondents. This could be majorly responsible for the young age of childbirth reported in the region. The level of perception of WRA to FP and contraceptives was quite mixed. The majority of respondents believes that FP contradicted their religious/cultural beliefs, thus portraying a negative perception of FP and contraceptives [19]) in Northern Nigeria and [20] in Bangladesh, both predominantly Muslim settings, have discussed the controversies surrounding Islamic beliefs and contraceptives, with both studies reporting majority of their sample abstaining from contraceptives due to religious reasons. Another controversial aspect of the perception discussion was that using contraceptives would make a woman promiscuous. This view has also been expressed by other researchers who reported similar findings in other African settings and how they impact negatively on the likelihood of using contraceptives [21,22].

## Conclusion

This study sought to provide this information on the knowledge and perceptions of women of reproductive age in Northwest Nigeria. The results show that the awareness of contraceptives and FP in the state continue to be on the rise with urban and educated residents being the most captured by awareness campaigns. However, the need to ensure that these campaigns reach the less educated who are more likely to live in rural backgrounds was also highlighted. Knowledge about contraceptives was also high indicating that channels currently in use may be getting the desired effect. Despite this positive position, the differentials observed within groups indicate that more needs to be done with regard to ensure disadvantaged groups have their knowledge on contraceptives improved. This is especially important among the lower age groups as improved knowledge among this group could result in their delaying their first pregnancy and hence, reduce maternal mortality. Perception of family planning was not entirely positive with issues surrounding the religious compatibility of FP and the fear of promiscuity coming out as key factors that impact negatively on the perception of WRA on FP and contraceptives. Community advocacy as well as more consultations with stakeholders will be necessary if the buy-in of the majority of the population in the region are to fully embrace contraceptive uptake.

### What is known about this topic

- Authors have confirmed that the major drivers of contraceptive uptake are demand and supply;
- Improving uptake is critical to achieving the sustainable development goals;
- Socio-cultural and religious factors are major influencers of contraceptive use.

### What this study adds

- This study reveals the specific perceptions of women of reproductive age on contraceptive uptake in North-west geopolitical zone of Nigeria;
- Contraception awareness and uptake is age-dependent, with older women of reproductive ages having a broader knowledge on contraception;
- Majority perceive contraception as a means to child spacing with only a few believing that it helps to limit family size.
